# Defining ambulatory care sensitive conditions for adults in Portugal

**DOI:** 10.1186/s12913-020-05620-9

**Published:** 2020-08-15

**Authors:** João Sarmento, João Victor Muniz Rocha, Rui Santana

**Affiliations:** 1 NOVA National School of Public Health, Public Health Research Center, Universidade NOVA de Lisboa, Av. Padre Cruz, 1600-560 Lisbon, Portugal; 2 Comprehensive Health Research Center, Universidade NOVA de Lisboa, Lisbon, Portugal

**Keywords:** Ambulatory care sensitive conditions, Delphi panel, Portugal

## Abstract

**Background:**

Ambulatory Care Sensitive Conditions (ACSCs) are health conditions for which adequate management, treatment and interventions delivered in the ambulatory care setting could potentially prevent hospitalization. Which conditions are sensitive to ambulatory care varies according to the scope of health care services and the context in which the indicator is used. The need for a country-specific validated list for Portugal has already been identified, but currently no national list exists. The objective of this study was to develop a list of Ambulatory Care Sensitive Conditions for Portugal.

**Methods:**

A modified web-based Delphi panel approach was designed, in order to determine which conditions can be considered ACSCs in the Portuguese adult population. The selected experts were general practitioners and internal medicine physicians identified by the most relevant Portuguese scientific societies. Experts were presented with previously identified ACSC and asked to select which could be accepted in the Portuguese context. They were also asked to identify other conditions they considered relevant. We estimated the number and cost of ACSC hospitalizations in 2017 in Portugal according to the identified conditions.

**Results:**

After three rounds the experts agreed on 34 of the 45 initially proposed items. Fourteen new conditions were proposed and four achieved consensus, namely uterine cervical cancer, colorectal cancer, thromboembolic venous disease and voluntary termination of pregnancy. In 2017 133,427 hospitalizations were for ACSC (15.7% of all hospitalizations). This represents a rate of 1685 per 100,000 adults. The most frequent diagnosis were pneumonia, heart failure, chronic obstructive pulmonary disease/chronic bronchitis, urinary tract infection, colorectal cancer, hypertensive disease atrial fibrillation and complications of diabetes mellitus.

**Conclusions:**

New ACSC were identified. It is expected that this list could be used henceforward by epidemiologic studies, health services research and for healthcare management purposes. ACSC lists should be updated frequently. Further research is necessary to increase the specificity of ACSC hospitalizations as an indicator of healthcare performance.

## Background

Ambulatory Care Sensitive Conditions (ACSCs) are health conditions-diagnoses for which timely and effective outpatient care can help to reduce the risks of hospitalization by either preventing the onset of an illness or condition, controlling an acute episodic illness or condition, or managing a chronic disease [1].

One of the first uses of ACSCs to evaluate a healthcare systems’ performance was described by Billings et al. [1] in 1993. Its objective was to evaluate the access to healthcare of New York’s indigent population. The theory behind this is as follows: if a certain condition-diagnose can be avoided or controlled, then the need for hospitalization could also be avoided. If the actions to avoid it can be taken in the ambulatory setting, then lack of access or effectiveness of ambulatory care should result in more hospitalizations.

This means that measuring ACSC hospitalizations can indirectly indicate how well the ambulatory care setting is performing. However, this information alone is not enough to accurately identify causes of poor performance. At least two largely different dimensions need to be taken into consideration. Firstly, individuals need to have access to ambulatory care in order to have certain conditions-diagnoses addressed. Secondly, the ambulatory setting needs to be effective.

When using ACSC hospitalizations as a performance indicator, lower number/rates of hospitalizations generally mean that ambulatory care is performing well. If numbers are higher it indicates that there could be a problem in access, effectiveness or both. This is the simplistic approach. The utility and information provided by ACSC as an indicator depends on further characteristics of the context in which they are measured. Nedel et al. [2] proposed major categories of influencing factors: Geographical characteristics; Sociodemographic characteristics and Model of care. The first category includes variables such as hospital proximity [3] or lower populational density and isolation [4], which increases the number of ACSC hospitalizations. Sociodemographic characteristics such as higher age, lower education, lower income or higher unemployment are also associated with higher rates of ACSC hospitalizations [4–8]. Comorbidities are an important risk factor [9], increasing the risk of hospitalization with each chronic condition or body system affected. Finally, the model of care has a significant impact on this indicator. Not only the organization and availability of healthcare providers has an impact, with lower rates where there is higher availability [4, 10], but also intrinsic characteristics of the model of care such as continuity of care plays important roles. Menec et al. [11] described that patients with more than 75% of consultations by the same family physician have fewer ACSC hospitalizations. Access to hospital beds also influences this indicator. If there is severe shortage of hospital beds then, even if the ambulatory care setting is not performing well, there will not be an increase in ACSC hospitalizations. On the other hand, if there is an excess of hospital beds, even if the ambulatory care setting is performing well, there might be an incentive to admit certain conditions and therefore increase ACSC hospitalizations [2].

ACSC hospitalizations are only measurable after they have occurred. However, when they occur the window of opportunity to avoid them in ambulatory care has already elapsed. Therefore, this means that measuring ACSC hospitalizations depends on the availability of hospital discharge data with enough detail. The key information is the most responsible diagnosis of each hospitalization, which if included in an ACSC list flags the hospitalization as potentially avoidable. Further information is also relevant to eventually detail other relevant determinants of hospitalization, such as patients’ age [3] and number of comorbidities [9].

Which conditions are sensitive to ambulatory care varies according to the scope of health care services and the context in which the indicator is used [2, 12]. Consequently, different methods to identify such hospitalizations have been developed worldwide. Different ACSCs lists have been developed since Billings et al. [1] in 1993, such as Caminal et al. [3], Brown et al. [13], Canadian Institute for Health Information (CIHI) [14], Page et al. [15], Purdy et al. [12], Alfradique et al. [16], Freund et al. [17], Sundmacher et al. [18] and the United States Agency for Healthcare Research and Quality (AHRQ) [19].

The process of defining ACSC lists usually starts with a literature review seeking to identify conditions with a potential relation to ambulatory care, followed by processes of discussion and validation with experts. This method aims to reach a consensus on which conditions are sensitive to ambulatory care according to the organization of care, disease mechanisms and prevalence, socioeconomic and cultural characteristics of the population and the patient course within the health system [2, 12].

Additionally, the inclusion of conditions usually follows specific criteria, being the Solberg and Weissman the most commonly used [20, 21]. These criteria include: (i) the existence of previous studies, (ii) clarity in the definition and coding of diagnoses, (iii) relevance for public health (a hospitalization rate of least 1/10,000 population), (iv) if the diagnosis is potentially avoidable by timely and effective ambulatory care and (v) the necessity of hospitalization.

The lists of ACSC developed in different countries are used by national and international institutions as a proxy for health care quality assessment. The WHO Regional Office for Europe produced a working document summarizing the evidence for using ACSC as an indicator for health care performance in the dimensions of access, quality, coordination and efficiency [22]. The OECD included hospitalizations for ACSC in the list of indicators for health care performance, under the argument that high hospitalization rates indicate possibilities of improving quality and reaching substantial cost savings, given that better primary care is provided [23, 24]. The NHS in England has been reporting information on ACSC as part of the NHS Outcomes Framework to monitor quality of care based on conditions sensitive to its primary health care [25] and has associated incentives to improve performance for certain ACSCs [26]. The AHRQ developed quality indicators for health care consisting of hospitalizations for ACSC [19], with conditions selected based on discussion of evidence in the dimensions of face validity, precision, minimum bias, construct validity, if it fosters true quality improvement and prior uses [27]. The ACSC list developed in Brazil is available for primary health care evaluation purposes [28]. In Portugal ACSS uses the rate of ACSC hospitalizations as an indicator of healthcare quality in vertical integrated healthcare units since 2016, and the ministry of health included this indicator, in a wider framework of evaluation, of primary care in 2017 [29].

The need for a country-specific validated list for Portugal has already been identified [30], however such tool has not been developed yet. Previous studies in Portugal have used different lists of ACSCs [31–34]. Some studies have indicated that, according to the list developed by Caminal et al. [3] for Spain, rates of ACSC hospitalizations have increased during the last years in Portugal [34–36]. However, according to the CIHI list [14], the rates have declined [32, 36]. The reason for such difference is in the methodology applied: while the Spanish list includes acute and chronic conditions, the CIHI list only contains chronic conditions. There is variation on rates of hospitalizations for ACSCs according to which conditions are selected and analysed [12, 30, 37, 38].

It was estimated that ACSCs represented 12.3% of all hospitalizations registered in mainland Portugal in 2013 [35]. These hospitalizations were concentrated in five main conditions: pneumonia, congestive obstructive pulmonary disease (COPD), heart failure, hypertensive heart diseases and kidney and urinary tract infections. Around 7.3% of the hospitalizations for ACSCs were of repeated patients [39].

Despite the complex framework of ACSCs determinants [2, 3], this is a valuable tool for health care assessment; it has been increasingly discussed not only at the academic level but also adopted by national health systems and international organizations as an indicator of performance [14, 19, 22, 40, 41].

The objective of this study was to develop a list of Ambulatory Care Sensitive Conditions for Portugal. A secondary goal was to identify ACSC hospitalizations based on the developed list.

## Methods

A modified Delphi panel approach was designed [1, 3, 16, 18], in order to determine which conditions can be considered ACSCs in the Portuguese adult population. It was web-based and asynchronous, being the answers submitted by each expert via a web-based form during a specified period of time. The experts were selected according to the following criteria:

- Specialist in General and Family Medicine (GP) or Internal Medicine (IM);
- Clinically active in the National Health Service;
- Preferably with an active academic collaboration;

We designed the mix of experts to be 75% GP and 25% IM. Being clinically active in the NHS was considered essential to assure the experts’ knowledge of the regularly available resources. The preferable active academic collaboration was included to increase the experts’ ability to understand the objectives and methods of this panel. The experts were selected, according to the defined criteria, by the most important scientific societies of each specialty, namely the Portuguese Association of General and Family Medicine (APMGF) and the Portuguese Society of Internal Medicine (SPMI). A total of 84 experts were invited to participate through an email invitation letter which included a description of the panels’ objective and methods. After agreeing to participate each expert was sent a link to a web-based form (institutional SurveyMonkey® account). The panel lasted from September 2017 to May 2018.

Firstly, a brief conceptual framework of ACSC and the difference between avoidable and adequate hospitalization was delivered. The first question then was “*In your opinion, and given the current legis artis, would you consider possible to avoid the need for hospital admission in Portugal in the following clinical conditions?*”. Experts were presented with a list of all previously identified ACSCs, for which a *yes/no/don’t know* box was available to be ticked as well as a commentary box, should the experts wish to discuss their answer. This list of conditions was compiled in a preparatory stage by searching *Pubmed* with the keywords “Ambulatory Care Sensitive Conditions”, “Avoidable admissions”, and “Avoidable hospitalizations”. The relevant results of this search are described in Table 1.

**Table 1:** Summary of relevant papers identified during the literature review to catalogue previously defined ACSCs

Author / Institution	Country	Year	Number ACSC	Method
Weissman, Gatsonis e Epstein [21]	USA	1992	12	Literature review + expert panel evaluation
Billings et al. / United Hospital Fund of New York [1]	USA	1993	28	Modified Delphi panel (6 physicians)
Sanderson e Dixon [42]	UK	2000	30	Modified Delphi panel (3 physician panels)
Davies et al. / Agency for Healthcare Research and Quality [27]	USA	2001	16 Prevention Quality Indicators (PQI)	Set of measures to identify ACSC. Based on literature review, empirical testing and consensus among expert panels.
Caminal et al. / Universidade Autónoma de Barcelona [3]	Spain	2001	35	Delphi Panel (44 experts)
Brown et al. / University of Oxford [13]	Canada	2001	27	Delphi panel (13 experts) + Modified Delphi panel (12 experts) + questionnaire panel (11 experts)
Purdy et al. / Bristol University [12]	UK	2009	19	Literature review.
Alfradique et al. [16]	Brazil	2009	20	Literature review + Experts consultation
Walker et al. [43]	Canada	2009	18	Administrative data + Expert consensus (9 experts)
Freund et al. [17]	Germany	2013	26	AHRQ + expert selection
Eggli et al. [44]	Switzerland	2014	19	Based on Purdy et al. + update of ICD-10 codes for specific conditions
Naumann, Augustin e Sundmacher [45]	Germany	2014	32	Based on Purdy et al. + 2 potentially relevant diagnoses
Sundmacher et al. [18]	Germany	2015	22	Literature review + Delphi panel (40 experts)

The second question was “*In your opinion, and given the current legis artis, would you consider possible to avoid the need for hospital admission in Portugal in any other clinical condition not previously mentioned?*”. Experts had an open answer box to fill. In each round, there was a final commentary box available for questions not directly related to specific conditions.

The consensus level was defined as 75% after a maximum of three rounds [3, 18]. The percentual level of consensus was calculated by dividing the frequency of answers by the number of respondents in each round, multiplied by 100. To allow for the items marginally over the consensus limit to still be discussed in the following rounds the clinical conditions with a level of consensus lower than 85% were included in the following rounds. After the first and second rounds, each expert was sent an individual spreadsheet with the aggregate results of the entire panel as well as his/her answers for each clinical condition. In the second and third rounds, each non-consensual condition was accompanied by a summary of the experts’ comments on the previous round. These summaries were convened after discussion between two researchers (JS and RS) and were anonymised. The clinical conditions not present in previous lists were identified by experts in the first round and still submitted to three rounds of discussion before the end of the panel.

After the experts validated the list, we identified hospitalizations for ACSC in Portugal in 2017, for the population aged 18 years or older, according to the principal diagnosis. We used the hospitalizations database provided by the Portuguese Central Administration of the Health System [*Administração Central do Sistema de Saúde- ACSS*]. We compiled two lists. The core list is composed by the conditions that fulfilled the criteria ii to iv proposed by Solberg and Weissman [20, 21]. Also an extended list is presented including all conditions agreed by the experts, despite not fulfilling all Solberg and Weissman criteria.

Costs of hospitalizations for ACSC were used to estimated potential healthcare savings associated to these events, according to the list obtained. Official hospitalization prices were used as proxy for costs, as done in previous studies in other countries [38, 46–48]. These prices are defined by Diagnosis Related Groups (all patients refined diagnosis related groups version 31.0) and severity of the condition, according to values published by the Ministry of Health [49].

## Results

Of the 55 invited GPs, 28 accepted to participate (50.9%) and 25 completed all rounds (89.3%). Regarding the Internal Medicine physicians, 29 were invited, 5 accepted to participate (17.2%) and 4 completed all rounds (80%).

Table 2 shows the panel’s results. In the first round, there was consensus (over 85%) in 22 of the 45 items (48.9%). In the second round, 7 further items achieved consensus. Finally, after three rounds, consensus (over 75%) was achieved for 34 items, leaving 11 non-consensual items (22.2%).

**Table 2:** Delphi Panel Results, pre-existing ACSCs

Condition	1st round	2nd round	3rd round
Acute myocardial infarction	22,86%	20,00%	48,28%
**Acute otitis media**	**97,14%**		
**Acute pharyngitis**	**97,14%**		
**Acute poliomyelitis**	62,86%	**86,67%**	
**Acute sinusitis**	**91,43%**		
**Acute skin infections**	**88,57%**		
**Acute tonsillitis**	**97,14%**		
Appendicitis with complications^a^	20,00%	10,00%	
**Asthma**	**88,57%**		
**Atrial fibrillation**	71,43%	76,67%	**82,76%**
**Chronic obstructive pulmonary disease / chronic bronchitis**	**88,57%**		
**Dehydration / Hydroelectrolytic changes**	80,00%	83,33%	**86,21%**
**Dementia**	62,86%	70,00%	**75,86%**
**Dental and oral cavity pathology**	**91,43%**		
**Diabetes mellitus (acute and chronic complications)**	**85,71%**		
**Diphtheria**	71,43%	**93,33%**	
Epilepsy and seizures	57,14%	53,33%	48,28%
**Folate deficiency anemia**	**91,43%**		
Gangrene	25,71%	43,33%	72,41%
**Gastroenteritis**	**91,43%**		
**Heart failure**	82,86%	**90,00%**	
**HBV acute infection**	77,14%	**90,00%**	
**HBV chronic infection**	**85,71%**		
**Hypertensive disease**	**97,14%**		
**Infectious parotitis**	82,86%	**86,67%**	
**Influenza**	**88,57%**		
**Iron deficiency anemia**	**91,43%**		
Ischemic myocardial disease	65,71%	70,00%	65,52%
**Measles**	82,86%	**86,67%**	
Meningitis by *H. influenzae*	48,57%	63,33%	48,28%
Nutritional deficiencies	82,86%	73,33%	68,97%
Pelvic inflammatory disease	65,71%	66,67%	55,17%
Perforated / bleeding digestive ulcer	37,14%	40,00%	65,52%
Periamygdaline abscess	60,00%	56,67%	51,72%
**Pneumonia**	**88,57%**		
Rheumatic fever	65,71%	76,67%	68,97%
**Rubella**	**88,57%**		
**Syphilis**	**97,14%**		
**Tetanus**	68,57%	**86,67%**	
**Tuberculosis**	77,14%	83,33%	**89,66%**
**Upper respiratory tract infection**	**97,14%**		
**Urinary tract infections**	**88,57%**		
**Urinary tract infections in pregnancy**	**85,71%**		
**Vitamin B12 deficiency anemia**	**91,43%**		
**Whooping cough**	80,00%	83,33%	**79,31%**

Table 3 shows the potentially new ACSCs suggested by the experts. Out of fourteen new conditions, four achieved consensus (over 75%) after three rounds and were included as ACSCs.

**Table 3:** Delphi Panel Results, newly proposed conditions

Condition	1st round	2nd round	3rd round
Abortion complications	46,67%	55,17%	34,48%
Alcoholic liver disease	70,00%	82,76%	72,41%
Bipolar disorder	70,00%	62,07%	58,62%
**Colorectal cancer**	50,00%	68,97%	**75,86%**
Hepatitis C	73,33%	82,76%	68,97%
HIV infection	76,67%	75,86%	68,97%
Lung cancer	50,00%	68,97%	62,07%
Melanoma	60,00%	65,52%	55,17%
Occupational accidents	56,67%	48,28%	48,28%
Occupational diseases	63,33%	58,62%	62,07%
Schizophrenia	66,67%	58,62%	55,17%
**Thromboembolic venous disease**^a^	56,67%	62,07%	**82,76%**
**Uterine cervical cancer**	56,67%	75,86%	**75,86%**
**Voluntary termination of pregnancy**	80,00%	79,31%	**86,21%**

^a^excluding pulmonary thromboembolism

Table 4 shows the number of hospitalizations for ACSCs in Portugal in 2017, for population aged 18 years or older, the hospitalization rate per 10,000 people and the ICD10 codes used for this identification. The conditions that fulfill the criteria ii to iv by Solberg and Weissman are in bold, and those compose the core list. In 2017, there were 847,609 hospitalizations of people aged 18 years or older; out of these, 133,427 were for ACSC (15.7% of all hospitalizations). This represents a rate of 1685 hospitalization per 100,000 adults. The most frequent diagnosis were pneumonia, heart failure, chronic obstructive pulmonary disease/chronic bronchitis, urinary tract infection, colorectal cancer, hypertensive disease atrial fibrillation and complications of diabetes mellitus.

**Table 4:** Portuguese ACSCs list, Core (in bold) and extended, ICD 10 codes and frequency of diagnosis, Portugal 2017

Condition	ICD 10	N of ACSHs	Hospitalization rate (per 10,000 population)
Acute otitis media^a^	H65.0, H65.1, H66.0	208	0.26
Acute pharyngitis^a^	J02	28	0.03
Acute poliomyelitis^a^	A80	1	0.00
Acute sinusitis^a^	J01	111	0.13
**Acute skin infections**	**L01-L04, L08**	**4158**	**5.25**
Acute tonsillitis^a^	J03	366	0.46
**Asthma**	**J45, J46**	**1513**	**1.91**
**Atrial fibrillation**	**I48**	**6218**	**7.85**
**Chronic obstructive pulmonary disease / chronic bronchitis**	**J40-J44, J47**	**10,476**	**13.23**
**Colorectal cancer**^**b**^	**C18-C20, C21.8**	**9664**	**12.29**
**Dehydration / Hydroelectrolytic changes**	**E86, E87.6**	**2169**	**2.73**
**Dementia**	**F01, F03, G30, G31.0, G31.8**	**1667**	**2.11**
**Dental and oral cavity pathology**	**A69.1, K02-K06, K08, K09, K12-K14**	**1566**	**1.98**
**Depression**	**F32, F33**	**3063**	**3.87**
**Diabetes mellitus (complications)**	**E10.0, E10.1, E10.5, E10.6, E10.8, E10.9, E11.0, E11.1, E11.5, E11.6, E11.8, E11.9, E12.0, E12.1, E12.5, E12.6, E12.8, E12.9, E13.0, E13.1, E13.5, E13.6, E13.8, E13.9, E14.0, E14.1, E14.5, E14.6, E14.8, E14.9, E15**	**5409**	**6.83**
Diphtheria^a^	A36	0	0.00
Folate deficiency anemia^a^	D52	35	0.04
**Gastroenteritis**	**A00 - A09**	**2412**	**3.05**
**Heart failure**	**I11.0, I13.0, I13.2, I50, J81**	**23,862**	**32.66**
HBV acute infection^a^	B16	22	0.03
HBV chronic infection^a^	B18.0, B18.1	58	0.07
**Hypertensive disease**	**I10, I11.9, I12, I13.1, I13.9, I15, I60, I61, I64, I67.4**	**6733**	**8.50**
Infectious parotitis^a^	B26	41	0.05
**Influenza**	**J9, J10, J11**	**842**	**1.06**
**Iron deficiency anemia**	**D50**	**1588**	**2.00**
Measles^a^	B05	4	0.00
**Obesity**	**E66**	**2635**	**3.33**
**Pneumonia**	**J13, J14, J15.3, J15.4, J15.7, J15.8, J15.9, J16.0 J16.8, J18**	**37,204**	**46.98**
Rubella^a^	B06	1	0.00
Syphilis^a^	A51 – A53	112	0.14
Tetanus^a^	A33 - A35	1	0.00
Thromboembolic venous disease^a,b^	I80, I82.2, I82.3, I82.8, I82.9	231	0.29
Tuberculosis^a^	A15-A17	778	0.98
Upper respiratory tract infection^a^	J06	254	0.32
**Uterine cervical cancer**^**b**^	**C53**	**929**	**1.17**
**Urinary tract infections**	**N10-N12, N30.0, N30.8, N30.9**	**10,347**	**13.07**
Urinary tract infections in pregnancy^a^	O23.0, O23.1, O23.2, O23.3, O23.4, O23.9	599	0.76
Vitamin B12 deficiency anemia^a^	D51	109	0.13
**Voluntary termination of pregnancy**^**b**^	**Z332**	**972**	**1.23**
Whooping cough^a^	A37	2	0.00

^a^ Do not fulfill criteria iii

^b^ Suggested by experts

Table 5 shows the estimated costs of hospitalizations for ACSC according to the core list. It was estimated that, in 2017, hospitalizations for ACSC in Portugal had a total cost of 352 million euros, indicating substantial healthcare costs might have been avoided. Around 48% of the total costs were for pneumonia and heart failure, and these two conditions were the most frequent. Hypertensive disease and colorectal cancer corresponded to 22% of the total costs: the average costs of each hospitalization for these conditions are the highest among all conditions (5533 and 4067 euros per hospitalization, respectively), given the complexity of these diseases.

**Table 5:** Costs of hospitalizations for ACSC

Condition	Cost (in euros)	% Total	Average cost per hospitalization (in euros)
Acute Skin Infections	7,235,584	2.06	1740
Asthma	2,253,453	0.64	1489
Atrial fibrillation	10,968,413	3.12	1764
Chronic obstructive pulmonary disease / chronic bronchitis	23,196,097	6.60	2214
Colorectal cancer	39,303,605	11.18	4067
Dehydration / Hydroelectrolytic Changes	3,798,308	1.08	1751
Dementia	4,248,041	1.21	2548
Dental and oral cavity pathology	2,362,121	0.67	1508
Depression	4,874,647	1.39	1591
Diabetes mellitus (complications)	12,961,718	3.69	2396
Gastroenteritis	4,283,792	1.22	1776
Heart failure	72,133,697	20.51	3023
Hypertensive disease	37,251,495	10.59	5533
Influenza	2,188,947	0.62	2600
Iron deficiency anemia	2,335,446	0.66	1470
Obesity	5,428,646	1.54	2060
Pneumonia	97,477,559	27.72	2620
Urinary Tract Infections	17,016,441	4.84	1645
Uterine cervical cancer	1,726,860	0.49	1858
Voluntary termination of pregnancy	624,279	0.18	642
**Total**	**351,669,148**	**100**	**2597**

Table 6 compares the list obtained by this study with some of the lists previously developed and in current use in other countries. It is important to mention that this table contains the broader identification of diagnostics, but specific ICD codes may vary between lists.

**Table 6:** Comparison of Portuguese list with previous lists

Conditions	Portugal	Alfradique	AHRQ	Billings	Caminal	CIHI	Freund	Page	Purdy	Sundmacher
Acute otitis media		X		X				X	X	X
Acute pharyngitis		X		X				X	X	X
Acute poliomyelitis					X		X	X	X	X
Acute sinusitis		X								X
Acute skin infections	X	X		X			X	X	X	X
Acute tonsillitis		X		X				X	X	X
Angina		X		X	X	X	X	X	X	
Asthma	X	X	X	X		X	X	X	X	X
Atrial fibrillation	X						X			
**Uterine cervical cancer**	X									
Chronic obstructive pulmonary disease / chronic bronchitis	X	X	X			X	X	X	X	X
**Colorectal cancer**	X									
Dehydration / Hydroelectrolytic Changes	X	X	X		X		X	X	X	X
Dementia	X									X
Dental and oral cavity pathology	X			X			X	X	X	X
Depression	X									X
Diabetes mellitus (acute and chronic complications)	X	X	X		X	X	X	X	X	X
Diphtheria		X			X		X	X	X	
Epilepsy		X		X		X	X	X	X	
Folate deficiency anemia										X
Gangrene					X		X	X	X	
Gastroenteritis	X	X		X			X			X
Heart failure	X	X	X	X	X	X	X	X	X	X
HVB acute infection		X					X	X	X	X
HVB chronic infection							X	X	X	X
Hypertension	X	X	X		X	X	X	X	X	X
Infectious Parotitis		X						X	X	X
Influenza	X						X	X	X	X
Iron deficiency anemia	X	X		X			X	X	X	X
Measles		X					X	X	X	X
Obesity	X									X
Pelvic inflammation		X		X	X		X	X	X	X
Perforated/bleeding ulcer		X			X		X	X	X	X
Pneumonia	X	X	X	X	X		X	X		X
Rubella		X					X	X	X	X
Syphilis		X			X					X
Tetanus		X		X	X		X	X	X	X
**Thromboembolic venous disease**	X									
Tuberculosis		X		X	X			X		X
Upper respiratory tract infection		X		X				X	X	X
Urinary Tract Infections	X	X	X		X				X	X
Urinary tract infections in pregnancy		X								X
Vitamin B12 deficiency anemia										X
**Voluntary termination of pregnancy**	X									
Whooping cough		X					X	X	X	X

## Discussion

This section will start with the methods’ discussion followed by the results’ discussion. To finalize we will briefly discuss this paper’s contribution to science and healthcare, as well as important underlying questions about using ACSCs as an indicator.

Regarding methods we have developed the list for the Portuguese context using the Delphi Panel method. All lists of ACSC have been developed using experts opinion to some extent, being the Delphi panel method the most frequently used [3, 16, 18]. The reason for this is the necessary evaluation of comprehensive and complex disease mechanisms and diagnostic/treatment pathways in order to determine if a condition can be avoided and/or treated in the ambulatory setting. No other method is described. The experts’ selection process is extremely important for the panels’ conclusions. Therefore, we intentionally mixed GPs with IMs since their perspectives are complementary. While the first are responsible for most ambulatory care in Portugal, therefore understanding the resources available in the ambulatory setting, the latter are the most responsible for hospital admission decisions and have the end-of-the-line perspective on the pathways that lead to an ACSC hospitalization. To ensure the experts quality we defined clear selection criteria and arranged for a peer-selection by the most relevant Portuguese scientific societies in each specialty. The difference in the invitation acceptance rate between GPs (50.9%) and IMs (17.2%) is very likely related to the higher familiarity with the concept of ACSC by GPs, once these are used as a performance indicator of primary care by local, regional and national authorities. The final mix of 86% GPs was higher than initially designed. However, the IMs were fairly active during the several rounds and positively contaminated the discussion on several items.

In terms of results the experts proposed fourteen new ACSCs having four conditions achieved consensus, namely uterine cervical cancer, colorectal cancer, thromboembolic venous disease and voluntary termination of pregnancy. For both type of cancers, the experts referred to the screening undertaken in primary care as the technology that might avoid the necessity of hospitalization. The reasoning is that early detected conditions might obliviate the need for more complex interventions that require hospital admission. This does not, however, mean that all cancers and therefore all admissions for cancer are avoidable. Regarding thromboembolic venous disease, experts identified the early diagnosis and treatment as capable of avoiding hospitalizations. Finally, voluntary termination of pregnancy was considered avoidable if effective and timely family planning is accessible. All of these conditions verified the relevance for Public Health criterion proposed by Solberg and Weissman (hospitalization rate higher than 1/10.000 hab) being colorectal cancer, in fact, the fifth highest rate of ACSC hospitalization in Portugal in 2017. Although these criteria have been widely accepted, we do not agree that Public Health relevance should be strictly reduced to a hospitalization rate. For this reason, we propose two lists of ACSC, the core list including only the conditions that verify the hospitalizations rate criterion, and the extend list where all conditions considered ACSC by experts are included.

It is also noteworthy that other four conditions strongly related to behavioural and lifestyle decisions were newly proposed and almost reached consensus, namely alcoholic liver disease, hepatitis C, HIV infection and lung cancer. The health promotion capacity of primary care was identified as the technology that might prevent the onset of these diseases and therefore the need for hospitalization. It is also relevant to discuss that the recent pharmacological innovation for the treatment of HIV and hepatitis C viruses infection might render the need for hospitalization growingly residual.

Another important discussion topic is the consensus in not considering appendicitis with complication an ACSC and the lack of consensus in further nine previously considered conditions. This fact elicits the discussion on the context specificities to which is important to adapt the ACSC lists. This may also reflect the limitation of using experts’ opinion to define ACSCs. In summary, the influx and efflux of conditions considered ACSC reflects the importance of regularly updating the lists. Nearly all the previous lists used in the comparison in Table 6 were developed more than 10 years ago. The constant development of knowledge, technology and healthcare design demands that the ACSC lists are updated more regularly.

Among the lists compared, the oldest one was proposed by Billings in 1993 [1]. Out of the 16 conditions proposed by Billings, only 7 are included in the list developed for Portugal, reflecting the need of updating lists. The lists proposed by the AHRQ and CIHI are the ones with less conditions included, while the expanded list obtained for Portugal is composed of 40 conditions that include acute, chronic and vaccine-preventable diseases.

The rates of hospitalization in this paper are in line with previous studies regarding the most frequent ACSCs in Portugal being pneumonia, COPD, cardiovascular diseases, urinary tract infection and diabetes [35, 50]. These conditions are included in most of the previous lists presented in Table 6.

Using the developed list, we identified that 15.7% of all hospitalizations were for ACSC. This is higher than what was found in previous studies, due to the inclusion of more conditions. Previous studies identified 4.4% [36], 9.9% [31] and 12.3% [35] between 2012 and 2015. These studies used different lists, therefore the differences between results.

To estimate the ACSC hospitalizations’ cost we used the best available proxy, as no billing system exists in Portugal, the “price” per hospitalization defined yearly by ACSS taking into consideration the diagnostic related groups methodology (all patients refined diagnosis related groups version 31.0) for the year 2017. Although this proxy might not represent the actual direct cost of ACSC it has been regularly used [38, 46–48]. The estimated 352 million euros represents 2% of the overall Portuguese healthcare expenditure [51]. According to the same source, the expenditure with public ambulatory care providers (excluding medication and diagnostic procedures) is 7.6%. This means that ACSC hospitalizations could cost yearly around 25% of what is spent with public ambulatory care providers. In another perspective, using the Gouveia et al. [52] estimation for the price per consultation in the Portuguese Primary Health Care (74.90€/per consultation including medication and diagnostic procedures), the estimated cost of ACSC hospitalization would translate into more than 4,6 million consultations in PHC, around 15% of all consultations that occurred in 2017 [53]. It is noteworthy that this exercise of cost-opportunity cannot be entirely fulfilled given that not all ACSC hospitalizations can be avoided.

The development of a validated list for the Portuguese context is important, as the use of a common methodology can standardize results, enhancing comparability within the country. The use of the country-specific list can also better reflect the health system organization and population characteristics of Portugal, therefore with a higher specificity to the Portuguese context. It does however hinder international comparability. Having validated an ACSC list for the Portuguese setting it is expected that henceforward official authorities and academic research use this list when measuring ACSC hospitalizations.

Finally, it is important to address some limitations inherent not only to this paper, but also to the concept and operationalization of ACSC as an indicator of access and quality of care.

The use of experts’ opinion in the definition of ACSCs is subject to several biases, as well as the process of translating conditions into diagnosis codes. Furthermore, the use of administrative databases intended for financial purposes and subject to coding quality variations also recommends precaution in the interpretation of the results herein described. Such limitation is inherent to studies using these databases, as recognized by previous studies [38, 54, 55]. However, these variations should not be relevant enough to compromise the overall picture, but should be taken into account when trying to zoom in to lower levels of aggregation. For example, while at the national and regional level this indicator is useful to identify Public Health priorities it might not be adequate at the local and individual level to evaluate specific providers’ access and quality once the aforementioned biases may cause an important lack of specificity. It is commonly discussed in ACSC studies that this indicator of quality of care does not apply to the individual experience of the patient and the medical practice [2, 56, 57].

The regional and local use should also take into account several determinants of ACSC hospitalizations, such as socioeconomic status, hospital distance and rurality [58–61], disease and multimorbidity prevalence [9], in order to achieve a necessary risk adjustment of results.

## Conclusion

We have developed a list of ACSCs for Portugal. This list includes conditions not identified in previous methodologies and excludes others previously identified thus increasing the specificity to the Portuguese context. Using this list to identify ACSC hospitalizations in Portugal, we have found a higher number of hospitalizations than what was estimated by previous studies. It is expected that this list could be used henceforward by epidemiologic studies, health services research and for healthcare management purposes. The identification of four new conditions contributes to the update of ACSC definition, highlighting that ACSC lists need to be frequently revisited and updated to keep up with technological and knowledge advances. Further research is necessary to increase the specificity of ACSC as an indicator of healthcare quality in smaller scales of analysis, such as individual or grouped providers. To increase this specificity, it will be necessary to determine how specific patient and context variables interact with each other in the pathway resulting in a ACSC hospitalization.

## Data Availability

The datasets used and/or analysed during the current study are available from the corresponding author on reasonable request.

## Abbreviations

ACSC: Ambulatory Care Sensitive Conditions
ACSS: Administração Central do Sistema de Saúde
APMGF: Portuguese Association of General and Family Medicine
COPD: congestive obstructive pulmonary disease
CIHI: Canadian Institute for Health Information
GP: General practitioners
HBV: Hepatitis B virus
HIV: Human immunodeficiency virus
ICD10: International Classification of Diseases 10th Version
IM: Internal Medicine physicians
JS: João Sarmento
SPMI: Portuguese Society of Internal Medicine
RS: Rui Santana
